# MicroRNAs Enhance Keratinocyte Proliferative Capacity in a Stem Cell-Enriched Epithelium

**DOI:** 10.1371/journal.pone.0134853

**Published:** 2015-08-06

**Authors:** Jong Kook Park, Wending Yang, Julia Katsnelson, Robert M. Lavker, Han Peng

**Affiliations:** 1 Department of Dermatology, Northwestern University, Chicago, Illinois, United States of America; 2 Rush University Medical Center, Chicago, Illinois, United States of America; University of Reading, UNITED KINGDOM

## Abstract

MicroRNAs are critical regulators of stem cell behavior. The miR-103/107 family is preferentially expressed in the stem cell-enriched corneal limbal epithelium and plays an important role in coordinating several intrinsic characteristics of limbal epithelial stem cells. To elucidate further the mechanisms by which miRs-103/107 function in regulating limbal epithelial stem cells, we investigate the global effects of miRs-103/107 on gene expression in an unbiased manner. Using antagomirs-103/107, we knocked down endogenous miRs-103/107 in keratinocytes and conducted an mRNA profiling study. We show that miRs-103/107 target mitogen-activated protein kinase kinase kinase 7 (MAP3K7) and thereby negatively regulate the p38/AP-1 pathway, which directs epithelial cells towards a differentiated state. Pharmacological inhibition of p38 increases holoclone colony formation, a measure of proliferative capacity. This suggests that the negative regulation of p38 by miRs-103/107 contributes to enhanced proliferative capacity, which is a hallmark of stem cells. Since miRs-103/107 also promote increased holoclone colony formation by regulating JNK activation through non-canonical Wnt signaling, we believe that this microRNA family preserves “stemness” by mediating the crosstalk between the Wnt/JNK and MAP3K7/p38/AP-1 pathways.

## Introduction

Stem cells are a population of relatively undifferentiated cells with the capability to self-renew and give rise to progeny (transit amplifying; TA cells). Such progeny can also proliferate but their capacity is finite and once exhausted, these TA cells differentiate into specialized cell types [1, 2]. Because of their high (infinite) proliferative capacity, stem cells play critical roles in tissue homeostasis and wound healing [3].

Cornea is comprised of three layers: epithelium, stroma, and endothelium. Like the epidermis, corneal epithelium functions as a dynamic barrier preventing entry of deleterious agents. Due to this protective function, the corneal epithelium is constantly shedding superficial cells, which must be replaced. Such a steady-state condition is, by definition, governed by stem cells, which are located in the basal layer of the limbal epithelium [4, 5], the transitional zone between cornea and conjunctiva. Limbal epithelial stem cells (LESCs) generate TA cells that migrate into the corneal epithelial basal layer [6–10]. These TA cells differentiate and migrate to the upper layers to replace the superficial cells that are continuously shed from the corneal epithelium during blinking. This steady-state process is critical for maintaining corneal epithelial homeostasis, and loss of LESCs because of eye diseases (e.g., ocular pemphigoid, Stevens-Johnson syndrome) or severe trauma (e.g., thermal and chemical burns) leads to corneal vascularization and opacification with severe visual loss [11, 12]. Therefore, it is vital and clinically significant to understand the behavior of LESCs and identify factors that regulate LESC physiology.

microRNAs represent a major class of regulatory noncoding small RNAs that negatively control their target gene expression via inhibition of translation or degradation of mRNAs. microRNAs have emerged as important regulators of stem cell potency, proliferation, differentiation, and survival [13–24]. For example, miR-205 is critical for regulation of epithelial stem cells [17, 19]. It controls stem cell proliferation and survival [19, 22], via targeting multiple negative regulators of the PI3K/Akt pathway including Frk, Inpp4b, Phlda3 and SHIP2 [19, 22]. miR-125b is a positive regulator of stem cell expansion and required for preserving a healthy stem cell pool by targeting Vdr, Trp53lnp1, Scarb1, and FGFR2 [20, 23]. In contrast, miR-203 has been suggested as a suppressor of epidermal stem cells [14, 15, 21]. miR-203 functions in promoting and maintaining epidermal stem cell differentiation through inhibition of its targets p63, Skp2 and Msi2 [14, 15, 21]. Thus microRNAs can regulate different characteristics of stem cells in the epidermis and hair follicle epithelium by modulating various downstream signaling pathways. Another well-studied stem cell-TA cell system is the limbal/corneal epithelium [4–10, 24–27]. Surprisingly, our understanding of how limbal epithelial stem cells are regulated by microRNAs is limited. We have begun to address this knowledge gap by isolating relatively pure populations of limbal basal (stem cells) and corneal basal (TA cells) epithelial cells using laser capture microdissection. Following microRNA expression profiling, we identified nine microRNAs that are preferentially expressed in the stem cell-enriched limbal basal epithelium [28]. Among them, we demonstrated that microRNAs-103/107(miRs-103/107) promote a slow cycling phenotype, enhance proliferative capacity, and maintain proper cell-cell communication in limbal epithelial stem cells [28].

In an effort to understand better how microRNAs affect limbal epithelial stem cell function, we employ gene function clustering analysis to connect the downstream target genes of limbal-preferred microRNAs to functional ontological pathways [28]. This unbiased analysis suggests that diverse processes are regulated by these limbal preferred microRNAs. Using bioinformatic analysis and biochemistry, we demonstrate that p38/AP-1 is a key downstream pathway of miRs-103/107 that contributes to the maintenance of stem cells.

## Materials and Methods

### Mice

All animals were obtained from the Charles River Laboratories. Whole eye globes were collected from wild-type female mice (Balb/c) immediately after sacrifice and rapidly embedded in OCT compound and stored at -80°C.

### Ethics Statement

All the procedures involving animals were approved by the Northwestern University Animal Care and Use Committee (NUACUC).

### Laser capture microdissection and microRNA qPCR

A PALM laser capture system (LCM; Zeiss) was used to isolate basal cells from limbal epithelium as previously described [28–30]. Taking advantage of the sharp demarcation between limbal and corneal epithelia based on the distinct morphological differences, ~40 cells/per limbus were captured avoiding the limbal/corneal junction as performed routinely by the laboratory [28–30]. Total cellular RNA from LCM-captured cells from individual mouse was isolated and purified using Qiagen miRNeasy kit (Qiagen). MicroRNA levels were measured using Exiqon's miRCURY LNA Universal RT miRNA PCR following the manufacturer's instructions.

### Known targets of microRNAs and Gene Ontology analysis

Known target gene lists of microRNAs were exported from miRTarBase, which is a database for experimentally validated microRNA-target interactions. Functional Annotation Clustering of known target genes by specific microRNAs was performed in DAVID Functional Annotation Bioinformatics Resources v6.7.

### Cell culture and antagomir treatment

To isolate and culture primary human limbal epithelial keratinocytes (HLEKs), cadaver donor corneas obtained from Eversight (Ann Arbor, MI) were explanted in CnT-20 media with supplements (CellnTech) [28]. For antagomir treatment, keratinocytes were incubated with 1μM antagomir, which can dramatically diminish the endogenous targeted microRNA [2, 28]. Antagomirs sequences are 5’- mU(*)mC(*)mA(*)mUmAmGmCmCmCmUmGmUmAmCmAmAmUmGmCmU(*)mG(*)mC(*)mU—Chol-3’ (antagomir-103), 5’-mU(*)mG(*)mA(*)mUmAmGmCmCmCmUmGmUmAmCmAmAmUmGmCmU(*)mG(*)mC(*)mU-Chol-3’ (antagomir-107), 5’- mG(*)mG(*)mC(*)mAmUmUmCmAmCmCmGmCmGmUmGmCmC(*)mU(*)mU(*)mA-Chol-3’ (irrelevant-antagomir; antago-124). Antago-124 was used as an irrelevant control since miR-124 is a neuronal specific microRNA [31] and undetectable in human limbal epithelium [24]. Consistently, antago-124 treatment in human limbal/corneal epithelia was not distinguishable biochemically and physiologically compared with untreated controls [22, 32]. “mN”: 2’-Omethyl-modified oligonucleotide. “(*)”: phosphorothioate linkage. “Chol”: cholesterol. All antagomirs were obtained from Dharmacon (Lafayette, CO).

### mRNA expression profiling and real time qPCR

HLEKs were treated with antagomir-103,-107 and irrelevant-antagomir and subsequently total cellular RNAs were isolated and purified by an mRNeasy kit (Qiagen, Hilden, Germany). Agilent Bioanalyser 2100 (Agilent, Santa Clara, CA, USA) was used to determine RNA quality. Samples with RIN >9 were used for the following experiment. Expression profiling was conducted using an Illumina chip (Illumina HumanHT-12 kit[28]). Differentially expressed genes were imported into DAVID Functional Annotation Bioinformatics Resources v6.7 and analyzed by Functional Annotation Clustering. To confirm the results of microarray profiling, cDNA was prepared using SuperScript II reverse transcription kit (Invitrogen). Real-time qPCR was performed on a Roche LightCycler 96 System using the Roche FastStart Essential DNA Green Master (Roche) according to the manufacturer’s instructions. Primer sequences used in this study were designed by Integrated DNA Technologies (IDT, Skokie, IL; S1 Table).

### Network Building

GeneGo program was used to build a network using the MetaCore Database and the Direct Interactions algorithm. This algorithm creates a network consisting only of the seed nodes and of their direct interactions; no other objects from the database are added. Seed nodes included: (i) the differentially expressed genes in antagomir-103/107-treated HLEKs (S2 and S3 Tables) and (ii) stem-cell-maintenance-related direct target genes of miRs-103/107 (S4 Table).

### Western Blotting

Western blots were performed as described previously [29]. The following antibodies were used at 1:1000 dilution: rabbit monoclonal anti-p-p38 (#4511, Cell Signaling Technologies), rabbit monoclonal anti-MAP3K7(#5206, Cell Signaling Technologies), rabbit monoclonal anti-p-MAP3K7(#9339, Cell Signaling Technologies), rabbit monoclonal anti-p-c-Jun(#2361, Cell Signaling Technologies), rabbit monoclonal anti-c-Jun(#9165, Cell Signaling Technologies), rabbit monoclonal anti-p38(#8699, Cell Signaling Technologies), and rabbit polyclonal anti-GAPDH (sc-25778, Santa Cruz Biotechnology).

### Luciferase reporter assay

The untranslated region (3’UTR) of MAP3K7 was ligated into the psiCHECK-2 vector (Promega) and luciferase reporter assay was conducted as described in detail previously [28]. Primer sequences for MAP3K7 3’UTR: sense, CTGCCTCGAGATTCTCTGGGACCGTTACAT; antisense, ATTAGCGGCCGCCAAATACATGAGAAAACAATCCAAGAATCA.

### Colony Formation assay

As previously described [28], HLEKs (200 cells per 100 mm dish) were seeded and incubated in FAD medium (DMEM/F12 media, FCS (10%), insulin (5 μg/ml), adenine (0.18 mM), hydrocortisone (0.4μg/ml), cholera toxin (10ng/ml), triiodothyronine(5μg/ml), 5μg/ml Human apo-transferrin, glutamine (4mM), penicillin-streptomycin (50 IU/ml), and Epidermal growth factor (10 ng/ml)) with mitomycin C-treated 3T3 feeder cells. To test whether inhibition of p38 can promote colony formation, HLEKs were pre-treated with p38 inhibitor SB203580 (10μM) or DMSO for 3 days and then plated in FAD medium. After 2 weeks, the cells were fixed and stained with methanol/crystal violet. Image J program was used to measure colonies.

### Statistical analysis

The significance of the differences between 2 groups was evaluated by an unpaired Student’s t test. The significance of the differences between 3 groups was tested by non-parametric one-way ANOVA.

## Results

**Fig 1:**
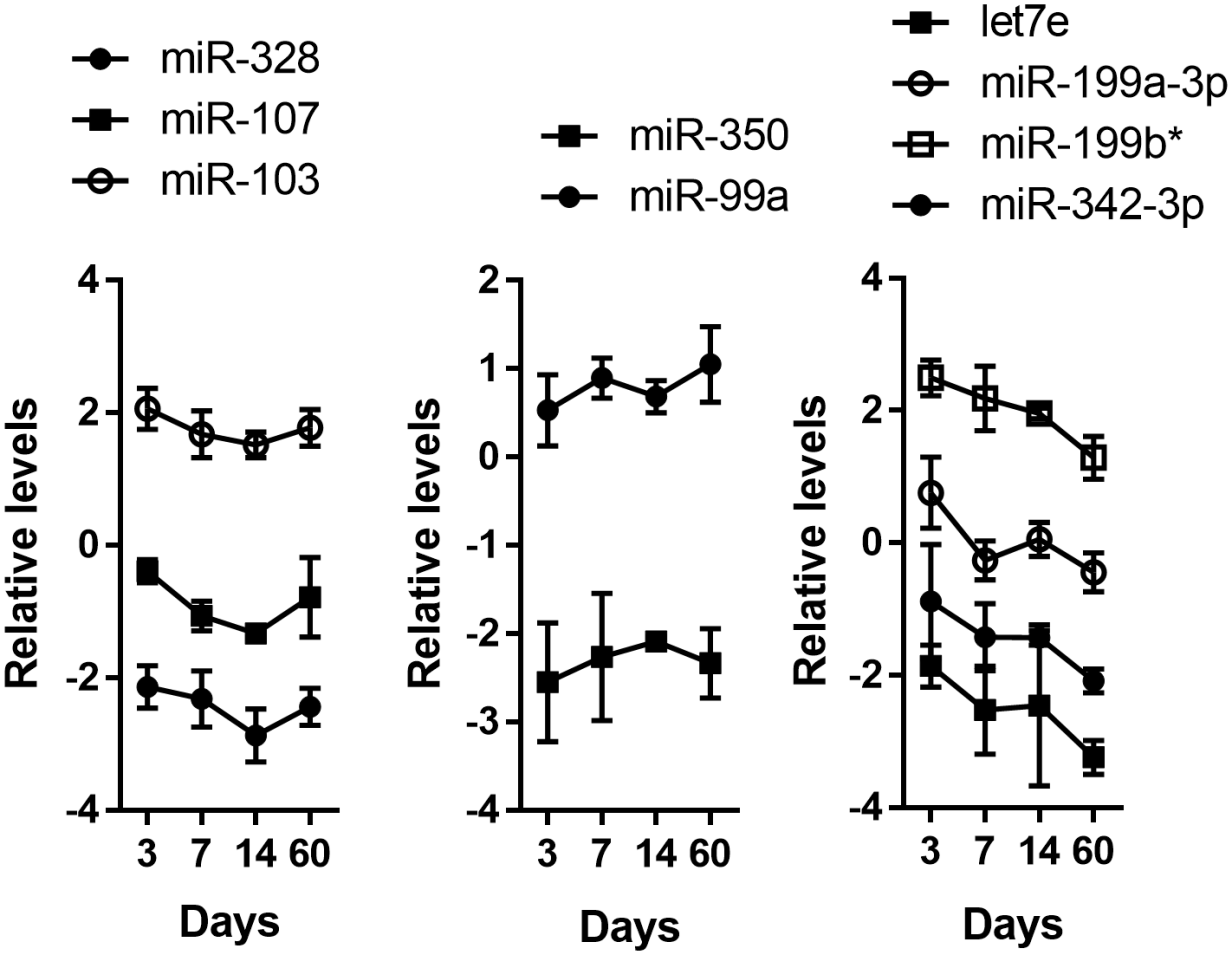
Expressions of limbal preferred microRNAs during postnatal eye development. MicroRNA qPCR analysis of miR-328, miR-103, miR-107, miR-350, miR-99a, let7e, miR-199a-3p, miR-199b*, and miR-342-3p levels in limbal epithelium at postnatal day 3, 7, 14, and 60. Values are means ± SD of three independent experiments.

Previously, we employed a high throughput microRNA profiling analysis to identify limbal-preferred microRNAs [28]. Among hundreds of microRNAs, we identified nine that were highly expressed in the stem cell-enriched limbal epithelial basal cells compared with TA cell-enriched central corneal epithelial basal cells [28]. To characterize their expression during mouse corneal development, we conducted real time qPCR for these nine microRNAs. RNAs were isolated from LCM-captured unperturbed limbal epithelial basal cells from the eyes of postnatal mice at day 3, 7, 14 and 60. Such time points represent different and critical developmental stages of limbal epithelium in post-natal life. For example (i) day 3 –K14 appears primarily in the basal cells [33, 34]; (ii) day 7 –there is a marked increase in corneal epithelial proliferation [35]; (iii) day 14 –is time of eye opening; and; (iv) day 60 –reflects a mature limbal epithelium [36]. We examined the expression of miR-184 and miR-31, which are two highly corneal-preferred microRNAs [22, 29, 37]. Consistently, levels of miR-184 and miR-31 were lower in the RNAs from isolated limbal epithelium compared with the RNAs from corneal epithelium (S1 Fig), confirming the relative purity of the isolated limbal epithelial cells. Interestingly, examination of the temporal expression of the nine miRNAs, revealed three types of patterns (Fig 1). Expression of let7e, miR-199a-3p, miR-199b*, and miR-342-3p decreased over time, whereas expression of miR-350 and miR-99a was relatively constant over the time periods analyzed. A third, more complex expression pattern was observed for miR-328, miR-103 and miR-107. Expression of these miRNAs decreased between day 3 and day 14 and then increased by day 60 (Fig 1). Such a “V” shape expression pattern implies a more dynamic regulation of miR-328, miR-103 and miR-107 indicative of multiple functions during corneal development.

**Fig 2:**
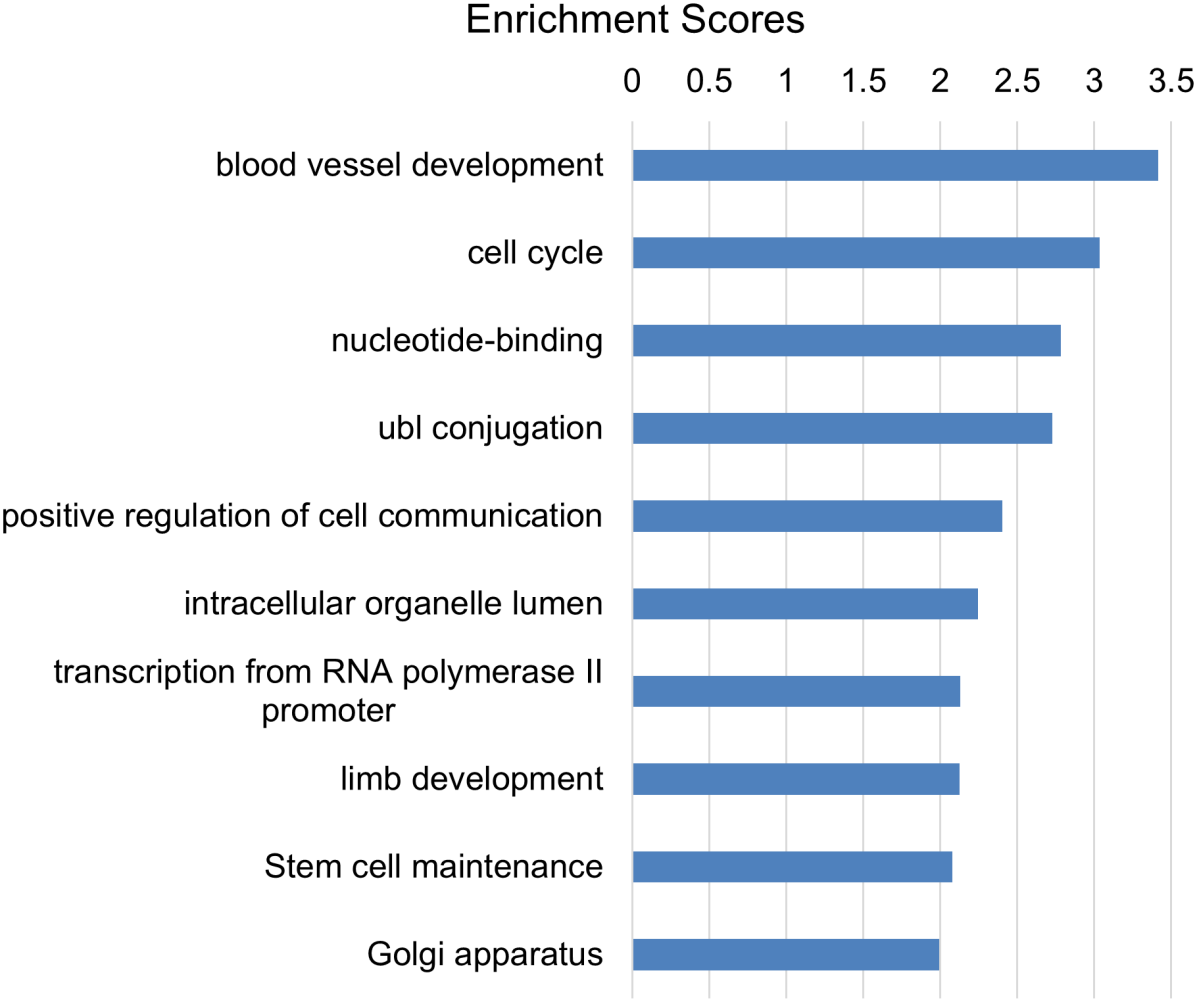
Functional Annotation Clustering analysis for known target genes of miRs-103/107. The known target gene list was exported from miRTarBase. The clustering analysis was performed by DAVID Functional Annotation Bioinformatics Resources v6.7. The rank is based on the Enrichment score, which represents mean p-value (in –log scale).

It’s not surprising that one microRNA can regulate multiple processes since a microRNA can target hundreds of genes [38–40]. With respect to limbal epithelial basal cells, we have demonstrated that miRs-103/107 target p90RSK2, Wnt3a, Nedd9 and PPTPRM and thus play roles in maintaining slow cycling, high proliferative capacity, and proper cell-cell communication [28], which are well-accepted characteristics of epithelial stem cells. Interestingly, our unbiased Functional Annotation Clustering analysis also indicated that miRs-103/107 functioned in the process of “stem cell maintenance” (Fig 2).

**Fig 3:**
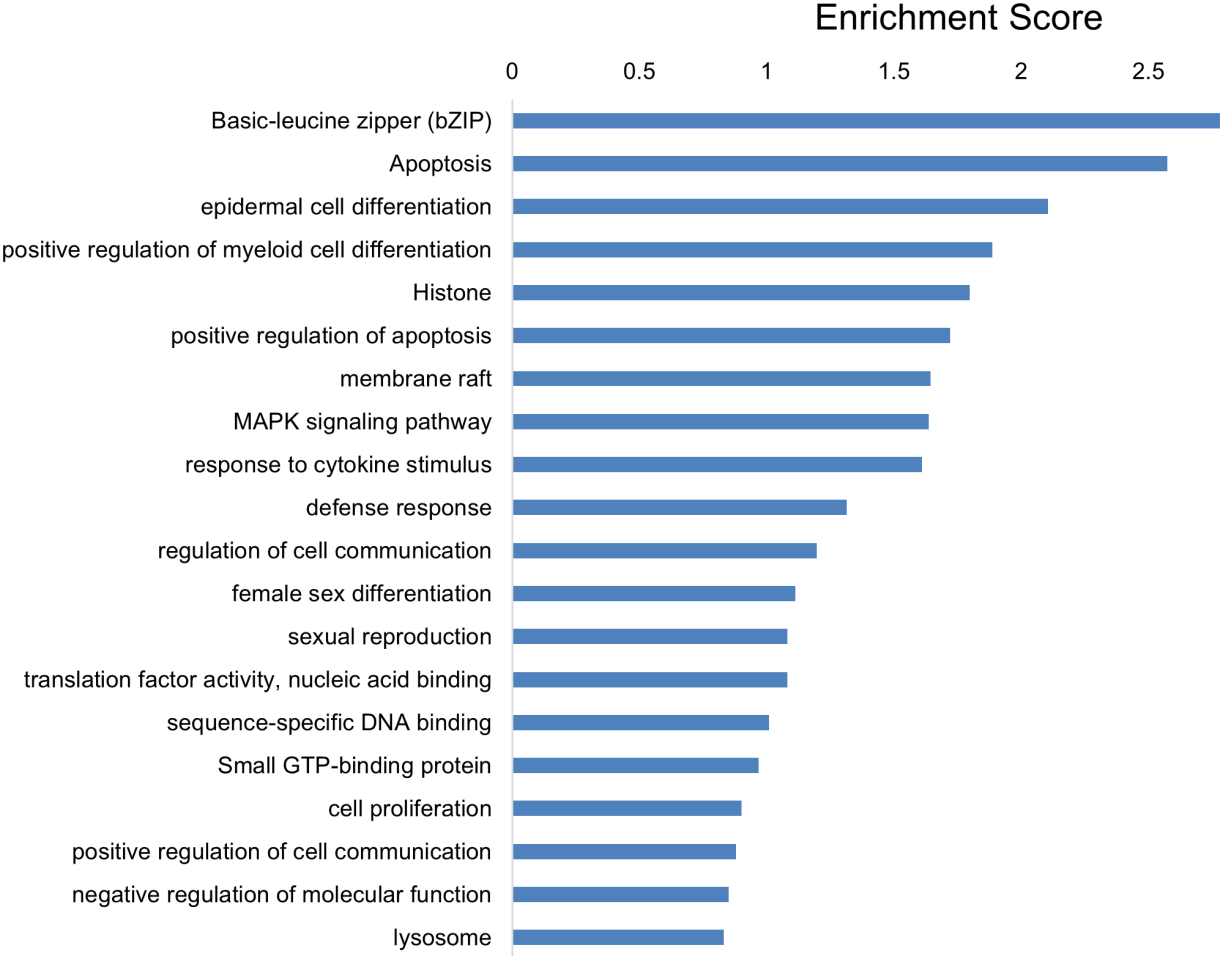
Functional Annotation Clustering analysis for differentially expressed genes in antago-103/107 treated HLEKs when compared with Ir-antago treated HLEKs. The clustering analysis was performed by DAVID Functional Annotation Bioinformatics Resources v6.7. The rank is based on the Enrichment score, which represents mean p-value (in –log scale).

miRs-103/107, which belong to a microRNA family, have identical sequences except for one nucleotide at the 3’-end and regulate overlapping targets [41]. Thus, to determine which processes were regulated by miRs-103/107, we examined the genes commonly regulated by this microRNA family [28]. We found that 123 genes were significantly changed following knock down of miRs-103/107 by antagomir treatment (adjusted p-value (false discovery rate) < 0.05 and fold change > 1.4; S2 and S3 Tables). Functional Annotation Clustering analysis revealed that these differentially expressed genes were associated with a variety of processes (Fig 3). Consistent with our previous study [28], this assay suggested that miRs-103/107 played important roles in epithelial cell differentiation, proliferation, and regulation of cell-cell communication.

**Fig 4:**
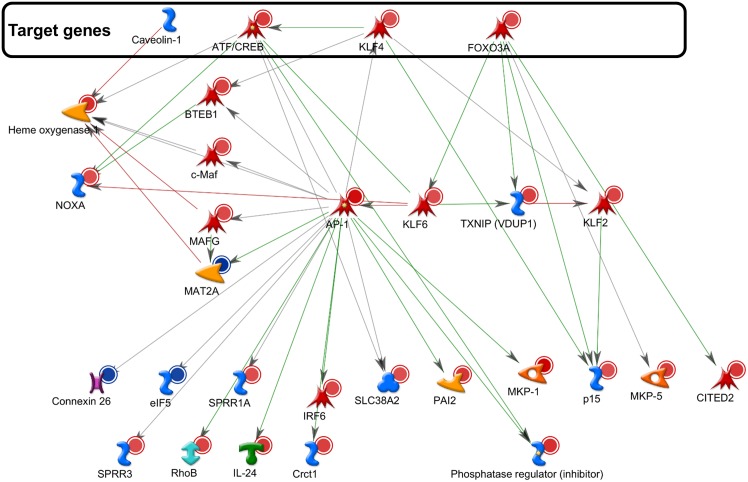
Networking analysis of miR-103/107-regulated genes. This network consists only of the seed nodes and of their direct interactions. Seed nodes included: (i) the differentially expressed genes in antagomir-103/107-treated HLEKs and (ii) miRs-103/107’s direct target genes that involve in stem cell maintenance. The genes in the box are known direct targets of miRS-103/107.

**Fig 5:**
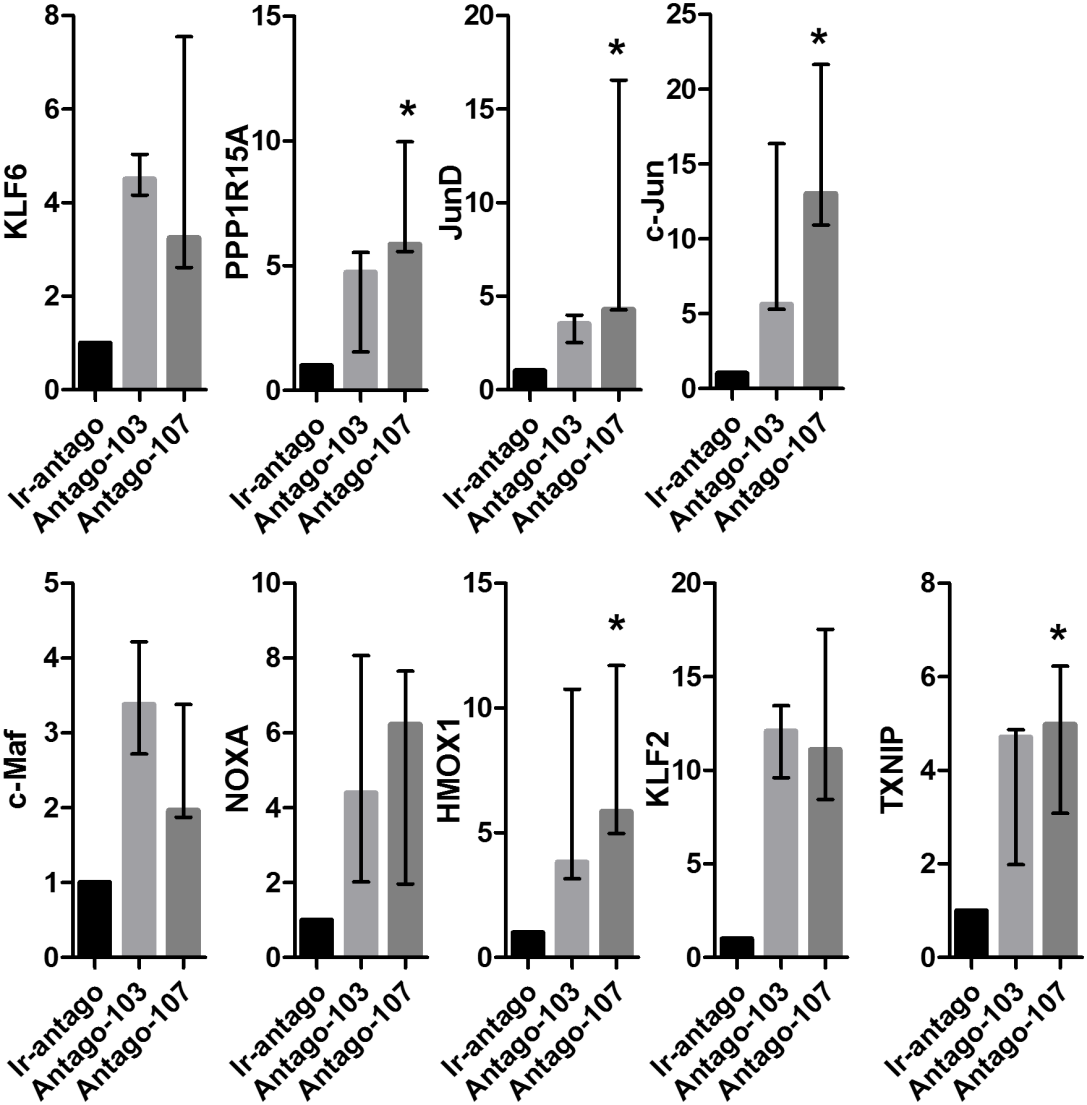
MiRs-103/107 negatively regulate AP-1 in HLEKs. Real time qPCR analysis of KLF6, PPP1R15A, JunD, c-Jun, c-Maf, NOXA, HMOX1, KLF2, and TXNIP levels in HLEKs that were treated with Ir-antago, Antago-103, or Antago-107. Values are median with range of four independent experiments. The significance of the differences between 3 groups was tested by non-parametric one-way ANOVA. *p<0.05.

To determine the potential downstream regulatory system that impacts on miRs-103/107 function in stem cell maintenance, the GeneGo program was used to build a network including: (i) the differentially expressed genes in antagomir-103/107-treated HLEKs and (ii) stem-cell-maintenance-related direct target genes of miRs-103/107 (Fig 4). The largest network formed by Direct interactions algorithm contained: (i) four stem-cell maintenance-related direct target genes of miRs-103/107 (CREB5, KLF4, FOXO3A, and caveolin-1; S4 Table); and twenty-five differentially expressed genes (Fig 4). To validate this data, we chose nine differentially expressed genes in this network and confirmed their alterations by antagos-103/107 using qPCR analysis (Fig 5). Among these differentially expressed genes, c-Jun and JunD, components of the AP-1 complex [42, 43], were upregulated by antagos-103/107 treatment (Fig 5).

**Fig 6:**
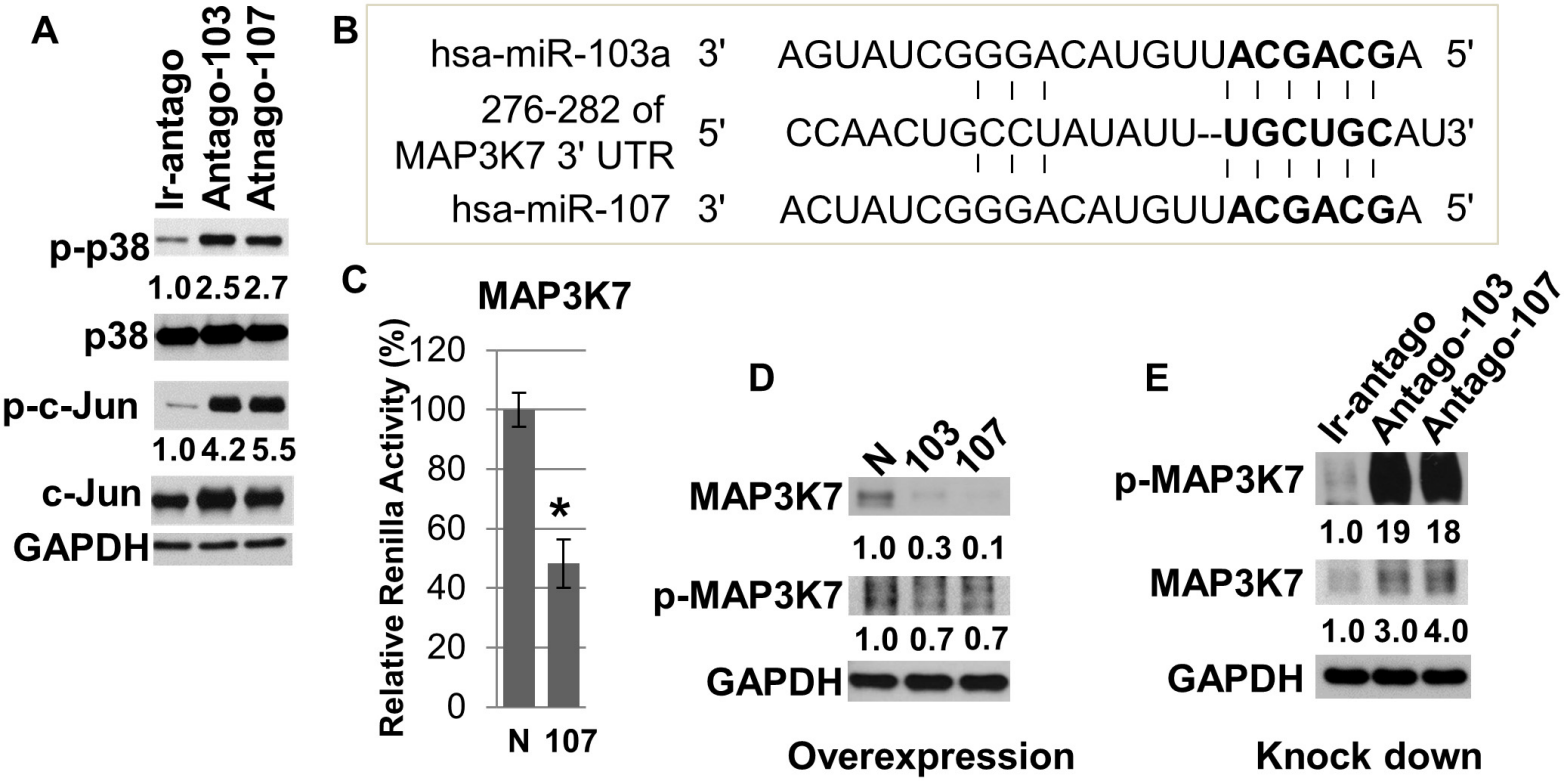
MiRs-103/107 negatively regulate p38/AP-1 pathway in HLEKs via directly targeting MAP3K7. (A) HLEKs were treated with Ir-antago, Antago-103 or Antago-107 for 24 hours; then lysates were immunoblotted against phospho-p38 (p-p38), p38, phosphor-c-Jun (p-c-Jun), c-Jun and GAPDH. (B) Schematic diagrams of miR-103/107 binding sites in the 3’UTR region of MAP3K7 mRNA. Bold: seed sequences. (C) Screening the interaction of MAP3K7 with miR-103/107 using the psiCHECKTM-2 vector harboring 3’ untranslated region (UTR) of MAP3K7. Constructs were co-transfected with either miR-control, or miR-107 into cells. Twenty four hour after transfection, firefly and renilla luciferase activities were measured using the Dual-Luciferase Reporter Assay System. N: precursor microRNA control, 107: precursor microRNA-107. T test was performed. *p<0.05. (D) Immunoblotting of endogenous total MAP3K7, phospho-MAP3K7 and GAPDH following over-expression of either pre-miR-negative control (N), pre-miR-103(103), or pre-miR-107(107). (E) HLEKs were treated with Ir-antago, Antago-103 or Antago-107 for 24 hours and lysates were immunoblotted for total MAP3K7, phospho-MAP3K7 and GAPDH. Numbers below the panels represent the normalized expression signal of proteins.

**Fig 7:**
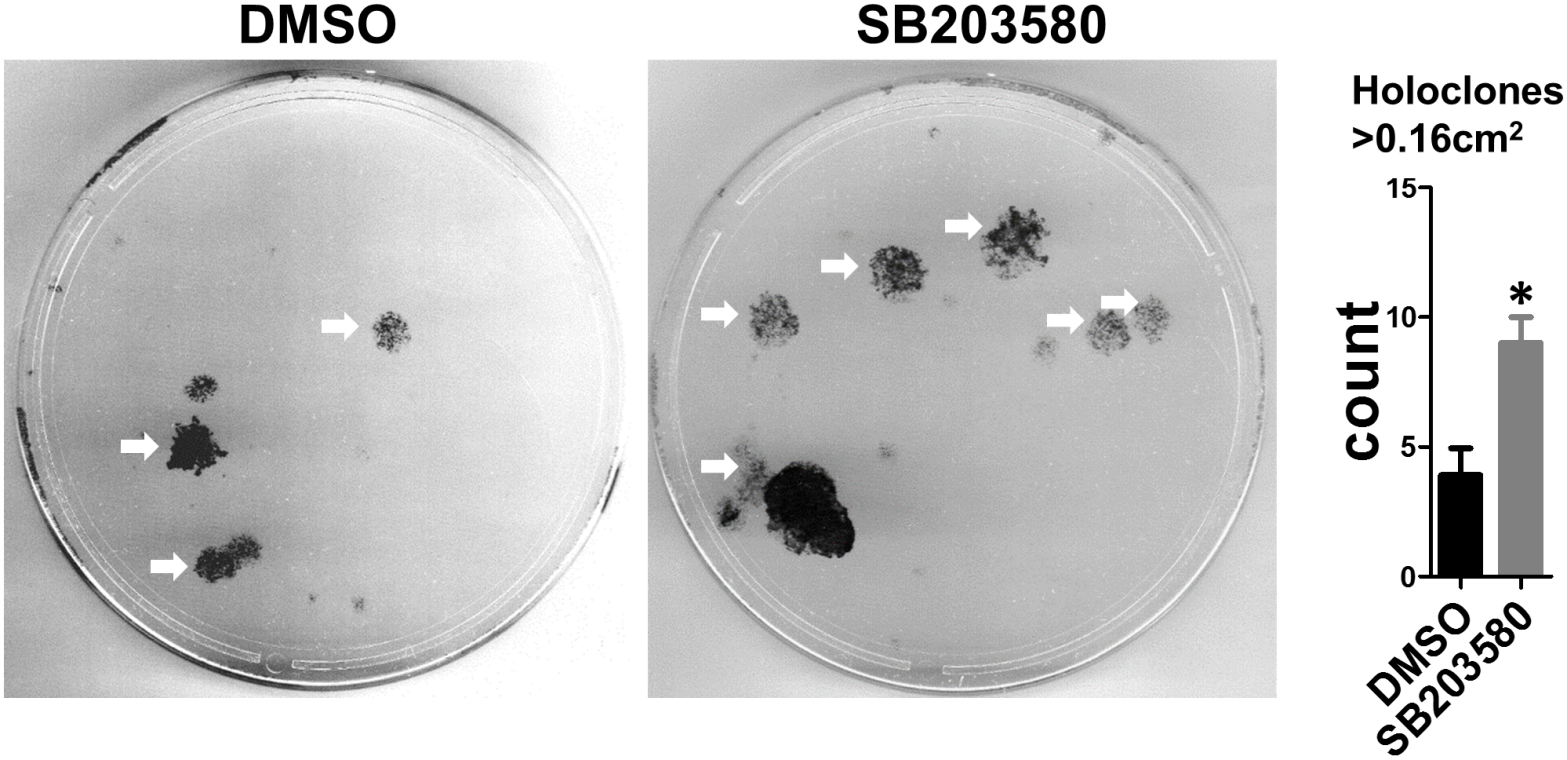
Inhibition of p38 increases holoclone formation. Representative holoclone forming assay of HLEKs following 3day pre-treatment with SB203580 (10μM; p38 inhibitor) or DMSO. SB203580-treated keratinocytes gave rise to significantly more holoclones than DMSO. 200 cells per plate were seeded for each treatment. White arrow: holoclone (colonies >0.16 cm^2^). T test was performed to calculate significance. *p<0.05.

It has been reported that activation of AP-1 and its well-established upstream regulator p38 is required for epidermal keratinocyte differentiation [44–47]. Since miRs-103/107 promoted stem cell phenotypes such as slow cycling and enhanced proliferative capacity [28], we explored the possibility that miRs-103/107 negatively regulated p38 and AP-1 to maintain such phenotypes. We used an antagomir approach [28, 29] to knock down miRs-103/107 in HLEKs and found that loss of miRs-103/107 dramatically increased phosphorylation of p38 and c-Jun (Fig 6A), suggesting that miRs-103/107 have an inhibitory effect on p38/AP-1 pathway. To investigate how this microRNA family negatively regulated p38, we used in silico and cell-based luciferase assays to find common targets, which are upstream of p38 pathway. We found that mitogen-activated protein kinase kinase kinase 7 (MAP3K7) was a novel target of this microRNA family (Fig 6B and 6C). Furthermore, immunoblotting showed that ectopic expression of miRs103/107 in HLEKs decreased levels of MAP3K7 and p-MAP3K7 (Fig 6D). Conversely, loss of miRs-103/107 by antagos-103/107 treatment significantly increased phospho-MAP3K7 and MAP3K7 levels compared to Ir-antago treatment (Fig 6E). These findings suggest that miRs-103/107 inhibit the p38 pathway via directly targeting the upstream activator MAP3K7. We then determined whether such inhibition of p38 in HLEKs affects proliferative capacity. To test this, we conducted colony formation assays to assess the ability to form holoclone colonies [48]. Holoclones are the colonies formed by keratinocytes with great proliferative capacity, and such keratinocytes are considered to be “stem cells” [48]. HLEKs, treated with the p38 inhibitor SB203580 (10μM) for 3 days, gave rise to significantly more holoclones colonies compared with DMSO treated HLEKs (Fig 7). This indicates that inhibition of p38 enhances keratinocyte proliferative capacity. Since the miR-103/107 family contributes to a stem cell phenotype by increasing the proliferative capacity of keratinocytes [28], our findings strongly suggest that miRs-103/107 targeting of MAP3K7 leads to attenuation of the differentiation-related p38 pathway, and thus helps to maintain the proliferative capacity of keratinocytes (stem cells).

## Discussion

We observed three different expression patterns over time among let7e, miR-199a-3p, miR-328, miR-350, miR-99a, miR-199b*, miRs-103/107 and miR-342-3p, which are limbal-preferred microRNAs [28]. Expression of miR-328, miR-103 and miR-107 decreased at day 7 (Fig 1) when keratinocytes are activated and there is a marked increase in corneal epithelial proliferation [35]. This negative association between the expression of these three microRNAs and the proliferative rates of keratinocytes suggests that miR-328, miR-103 and miR-107 may have an inhibitory effect on proliferation. In support of this idea, we have shown that miRs-103/107 are negative regulators of cell cycle [28].

MiRs-103/107 increase the proliferative capacity of keratinocytes by targeting Wnt3a [28], suggesting a positive role of this microRNA family in stem cell maintenance. Inhibition of Wnt3a bypasses JNK-mediated repression of YAP1 [28], a determinant of epithelial stem cell proliferative capacity [49, 50], and promotes a stem cell phenotype [28]. In addition to YAP1, JNK also phosphorylates JunD and c-Jun [51], components of the AP-1 complex. Such phosphorylation increases activity of JunD and c-Jun [51]. Interestingly, inhibition of either JNK or the AP-1 complex represses keratinocyte differentiation [45–47]. Since depletion of miRs-103/107 activated JNK [28] and increased expression of JunD and c-Jun (Fig 5A), we believe that inhibition of the JNK/AP-1 pathways is another mechanism by which this microRNA family promotes an undifferentiated phenotype in LESCs. In addition to JNK, loss of miRs-103/107 also increased activity of p38, another MAPK (Fig 6A). Similar to JNK, p38 phosphorylates c-Jun and activates its DNA binding activity [52]. Interestingly, activation of the p38/AP-1 pathway is required for keratinocyte differentiation [44–47]. Taken together, our findings suggest that AP-1 and the two MAPKs form a key regulatory network, by which miRs-103/107 may contribute to an undifferentiated state in LESCs. In support of this idea, c-Jun positively regulates expression of keratinocyte differentiation markers such as transglutaminase 1 [53, 54], loricrin [55, 56], and involucrin [54]. Moreover, JunD increases expression of Cystatin A [57], a gene specifically expressed in terminally differentiated keratinocytes [58].

Interestingly, MAP3K7, an upstream activator of MAPK/Jun pathway, is a direct target of miRs-103/107 (Fig 6B–6E). In addition to activating p38 and JNK, MAP3K7 also can activate IkappaB kinases, leading to the activation of NF-kappaB [59, 60]. Since NF-kappaB plays a role in limbal epithelial growth in vitro [61], it will be interesting to investigate whether NF-kappaB pathway contributes to the increased keratinocyte proliferative capacity by miRs-103/107.

Besides JunD and c-Jun, miRs-103/107 also affected other regulators of differentiation (Figs 4 and 5). For example, human Krüppel-like transcription factor 6 (KLF6) has been implicated in corneal physiology and integrity by positively regulating keratin12 [62], a keratin associated with corneal epithelial differentiation [5]. Embryonic stem cells lacking KLF6 failed to commit to a hematopoietic differentiation pathway and showed delayed expression of differentiation markers, Brachyury, Klf1 and Gata1 [63]. Furthermore, miRs-103/107 negatively regulated expression of c-Maf, which is a leucine zipper-containing transcription factor and is required for crystallin expression [64, 65], a marker of lens fiber cell differentiation [66]. Loss of c-Maf markedly impaired differentiation of lens fiber cells [64, 65], resulting in a severe developmental defect in lens. However, the role of c-Maf in limbal/corneal epithelia remains unknown [64].

## Conclusions

In summary, much progress has been achieved in understanding how miRNAs function in a variety of tissues [67, 68] Since stem cells are a frequent site of neoplastic transformation [69–71], it is not surprising that much attention has been directed at understanding microRNAs in the context of cancer stem cells [72–77]. However, how microRNA regulate resting or “normal” stem cells, remains understudied. Our study begins to unravel the complexities underlying how a particular microRNA family influences limbal epithelial stem cell behavior as well as how this family is regulated.

## Supporting Information

S1 Fig Differential expression of miR-184 and miR-31 in limbal and corneal epithelia. MicroRNA qPCR analysis of miR-184 and miR-31 levels in corneal and limbal epithelia at postnatal day 3, 7, 14, and 60. Values are means ± SD of three independent experiments.
(TIF)

S2 Fig Full western blot pictures with molecular weight standard. (TIF)

S1 Table Primer sets for real time PCR. (XLSX)

S2 Table Genes that were upregulated by antago-103/107 treatment in HLEKs. (XLS)

S3 Table Genes that were downregulated by antago-103/107 treatment in HLEKs. (XLS)

S4 Table Known direct target genes of miRs-103/107 that were associated with stem cell maintenance. (XLSX)
