# Supplementary figures and images for: MicroRNAs Enhance Keratinocyte Proliferative Capacity in a Stem Cell-Enriched Epithelium
Source: PLoS One. 2015 Aug 6;10(8):e0134853. doi: 10.1371/journal.pone.0134853 (PMC4527697; doi:10.1371/journal.pone.0134853)

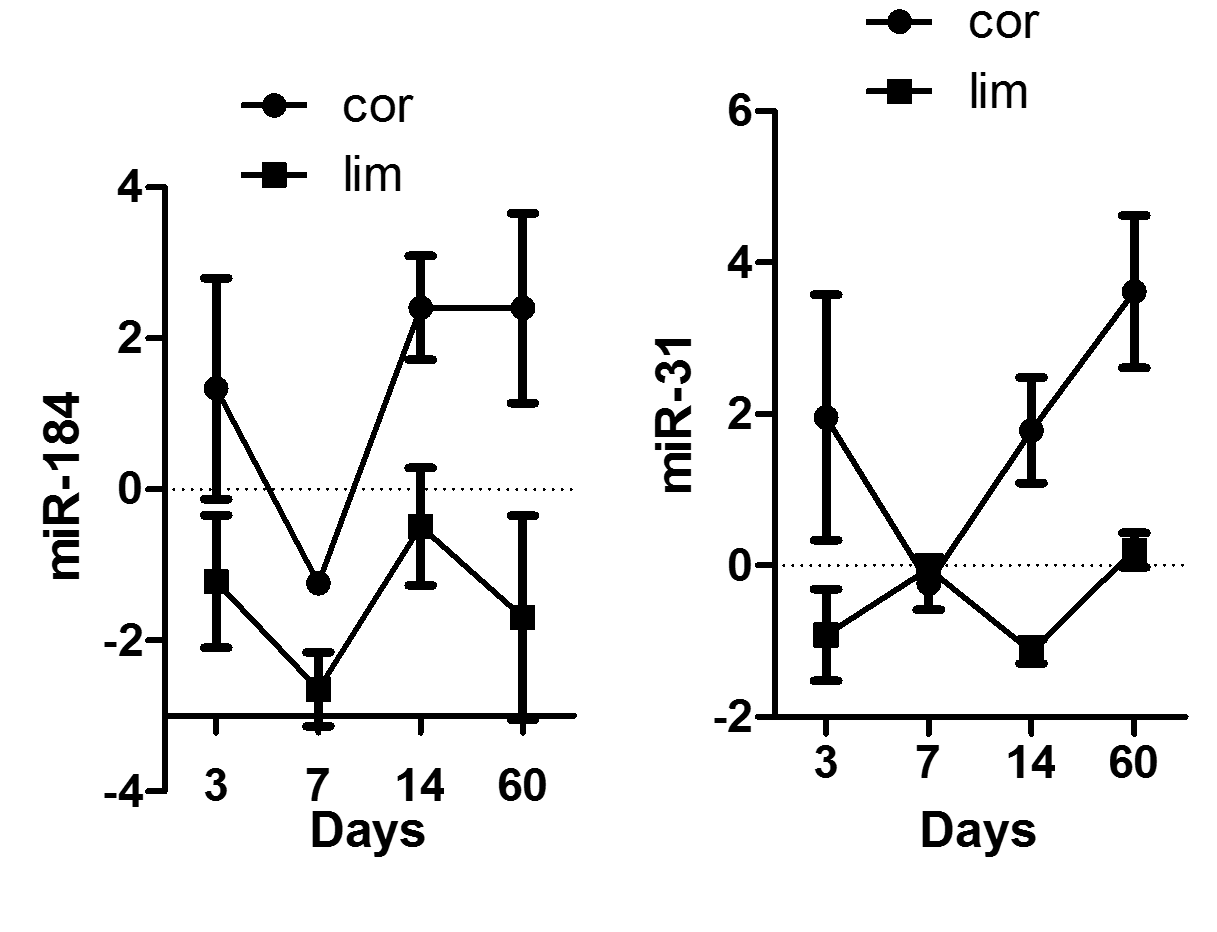

Supplement: S1 Fig — MicroRNA qPCR analysis of miR-184 and miR-31 levels in corneal and limbal epithelia at postnatal day 3, 7, 14, and 60. Values are means ± SD of three independent experiments. (TIF) [file pone.0134853.s001.TIF]

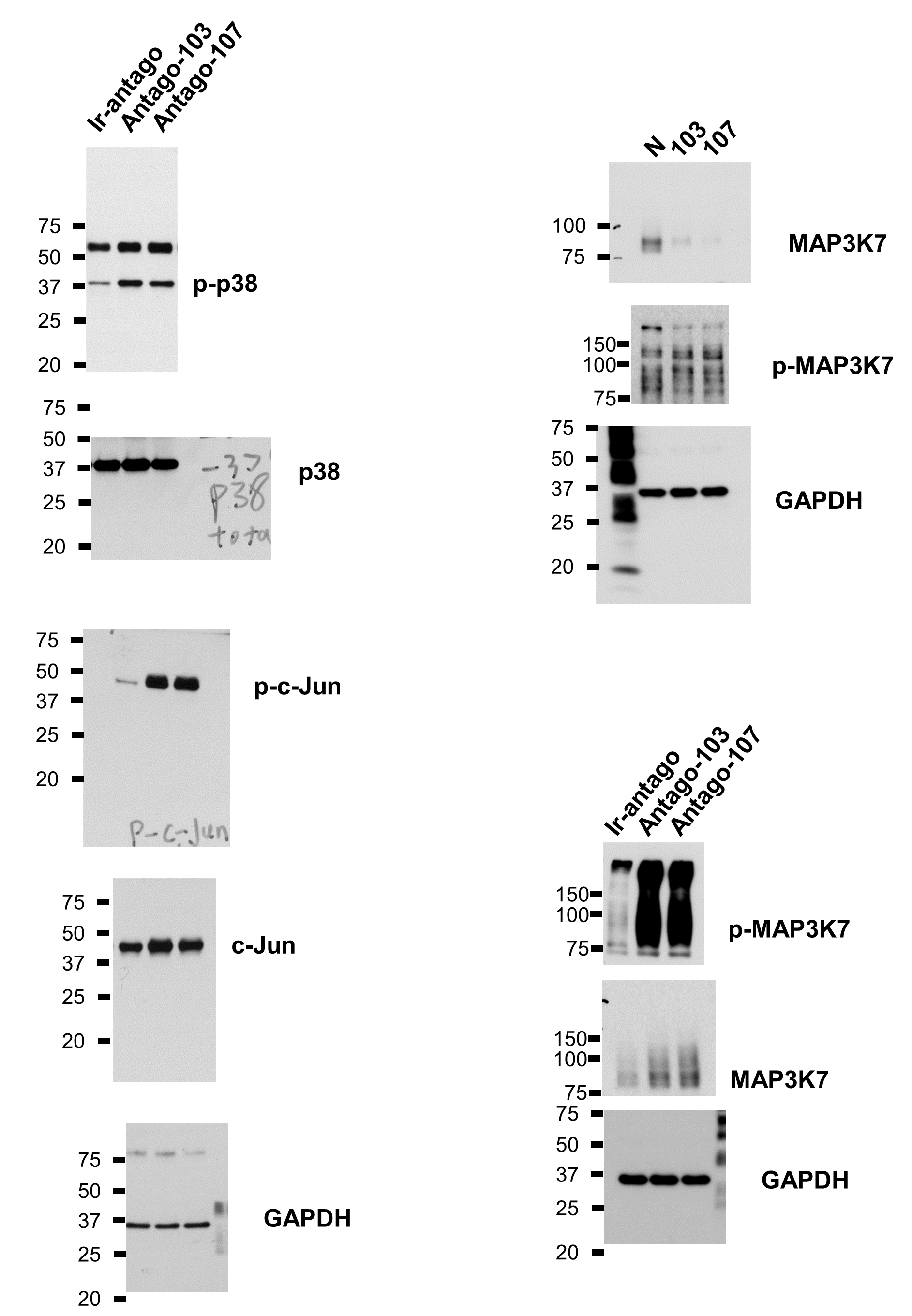

Supplement: S2 Fig — (TIF) [file pone.0134853.s002.TIF]
